# Accounting for imperfect detection of groups and individuals when estimating abundance

**DOI:** 10.1002/ece3.3284

**Published:** 2017-08-08

**Authors:** Matthew J. Clement, Sarah J. Converse, J. Andrew Royle

**Affiliations:** ^1^ U.S. Geological Survey Patuxent Wildlife Research Center Laurel MD USA; ^2^ Arizona Game and Fish Department Phoenix AZ USA; ^3^ U.S. Geological Survey Washington Cooperative Fish and Wildlife Research Unit School of Environmental and Forest Sciences (SEFS) and School of Aquatic and Fishery Sciences (SAFS) University of Washington Seattle WA USA

**Keywords:** abundance, aerial surveys, distance sampling, double observer, grouped animals, mark‐recapture‐distance‐sampling, N‐mixture models

## Abstract

If animals are independently detected during surveys, many methods exist for estimating animal abundance despite detection probabilities <1. Common estimators include double‐observer models, distance sampling models and combined double‐observer and distance sampling models (known as mark‐recapture‐distance‐sampling models; MRDS). When animals reside in groups, however, the assumption of independent detection is violated. In this case, the standard approach is to account for imperfect detection of groups, while assuming that individuals within groups are detected perfectly. However, this assumption is often unsupported. We introduce an abundance estimator for grouped animals when detection of groups is imperfect and group size may be under‐counted, but not over‐counted. The estimator combines an MRDS model with an N‐mixture model to account for imperfect detection of individuals. The new MRDS‐Nmix model requires the same data as an MRDS model (independent detection histories, an estimate of distance to transect, and an estimate of group size), plus a second estimate of group size provided by the second observer. We extend the model to situations in which detection of individuals within groups declines with distance. We simulated 12 data sets and used Bayesian methods to compare the performance of the new MRDS‐Nmix model to an MRDS model. Abundance estimates generated by the MRDS‐Nmix model exhibited minimal bias and nominal coverage levels. In contrast, MRDS abundance estimates were biased low and exhibited poor coverage. Many species of conservation interest reside in groups and could benefit from an estimator that better accounts for imperfect detection. Furthermore, the ability to relax the assumption of perfect detection of individuals within detected groups may allow surveyors to re‐allocate resources toward detection of new groups instead of extensive surveys of known groups. We believe the proposed estimator is feasible because the only additional field data required are a second estimate of group size.

## 1 INTRODUCTION

Although many methods exist for estimating animal abundance when probability of detection is <1, grouped animals present unique challenges. Two common approaches to survey grouped populations include distance‐sampling (Buckland et al., 2001) and double‐observer (Cook & Jacobson, 1979) methods. Both approaches assume that groups are detected independently and that all individuals within a group are detected, conditional on the group being detected. However, both logic and experience suggest that some individuals will be missed (Cogan & Diefenbach, 1998; Fleming & Tracey, 2008; Graham & Bell, 1989), thereby negatively biasing abundance estimates. Although this negative bias is well known, “there is no obvious correct approach” for eliminating the bias (Laake, Dawson, & Hone, 2008). Therefore, a practical approach to accounting for both imperfect detection of groups, and individuals within those groups, could improve abundance estimates across a variety of applications.

Although originally developed as independent approaches, distance‐sampling and double‐observer methods are sometimes used in tandem to improve abundance estimates (Borchers, Zucchini, & Fewster, 1998). Double‐observer models rely on detection history data, which represent which groups (or individuals, for non‐grouped animals) were seen by which observers. For example, a group may have a detection history of 11, 01, or 10, indicating the group was seen by both observers, only the second observer, or only the first observer, respectively. Intuitively, a data set with many 11 detection histories indicates a high detection probability, whereas many 0s in the detection histories indicate a low detection probability. However, double‐observer methods can be biased under detection heterogeneity, that is, unmodeled variation in detection probability among groups, a common occurrence in double‐observer surveys (Barker, 2008). Distance sampling relies on distance‐to‐transect data for each observed group, and the reasonable assumption that detection declines with distance (Buckland et al., 2001). However, if detection is <1 on the survey transect, abundance estimates can be biased (Buckland et al., 2001). An approach called mark‐recapture‐distance‐sampling (MRDS) combines both data types and model likelihoods to reduce the biases that arise when each method is used independently (Laake & Borchers, 2004; Laake et al., 2008). However, the MRDS approach does not address imperfect detection of individuals within groups; the common assumption is that all individuals within detected groups are counted.

**Figure 1:**
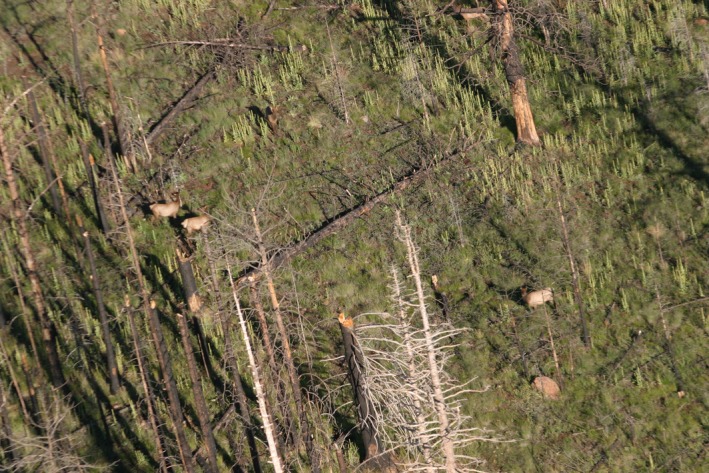
A view of elk (*Cervus elaphus*) during an aerial survey. Three elk are readily visible, while one blends into the background and one is partially obscured by a tree branch

A representative example of the sampling situation that motivated our investigation of methods to account for missed individuals in detected groups is the regular surveys of large ungulates, including elk (*Cervus elaphus*), conducted by the Arizona Game and Fish Department (AZGFD). Biologists applying the double‐observer method to elk surveys were concerned about the accuracy of group‐size estimates. Trees, shadows, ground cover coloration, light conditions, animal behavior, calves obscured by adults, and the natural camouflage provided by counter‐shaded dun pelage can make it difficult to count elk after a group has been detected (Figure 1). Arizona Game and Fish Department biologists were concerned that these visual estimation difficulties might bias abundance estimates, but there were no estimators available to address this concern. Therefore, we resolved to try to develop an abundance estimator that could account for imperfect detection of both groups and individuals.

Here, we propose a method to account for imperfect detection of individuals within groups by incorporating N‐mixture models (Royle, 2004) into MRDS models. N‐mixture models are hierarchical models that rely on repeated counts of individuals to estimate detection probability and abundance. Under our approach, observers make independent counts of each observed group, in addition to the detection history and distance‐to‐transect data collected for an MRDS model. By combining the three distinct sampling ideas into a unified hierarchical model, we can obtain unbiased abundance estimates even when group size is measured with error. We expect this data collection to be achievable because the only difference from current MRDS sampling design is that each observer should independently count and record group size, instead of conferring and recording a single group‐size count (e.g., Laake et al., 2008). We posit that such data are routinely collected but often summarized after data collection to discard auxiliary information on group size (Conroy et al., 2008). However, because the N‐mixture component introduces an assumption that individuals are not double‐counted, survey protocols may need to be refined to meet this assumption.

We present a new estimator for abundance of grouped animals when detection of groups is imperfect and group size may be under‐counted. We introduce the sampling situation and the formal model, and then demonstrate its application to simulated data. We also demonstrate the bias associated with a reduced estimator that assumes group size is recorded without error.

## 2 SAMPLING DESIGN AND MODEL DESCRIPTION

Our goal is to estimate animal abundance when groups of animals are imperfectly detected and when group size may be under‐counted. Our approach is an extension of available MRDS models. Accordingly, the proposed sampling design is a slight modification of the MRDS sampling design (Burt, Borchers, Jenkins, & Marques, 2014). As with MRDS sampling, we propose that multiple surveyors conduct distance sampling, either traversing a line transect or conducting point counts. The surveyors should observe groups of animals and record which groups were seen by which surveyors, and the perpendicular distance from the groups to the transect line. The only survey modification that we propose is that each observer should record an independent count of group size, rather than a unified count for all observers.

The observed counts of animals are likely to underestimate true abundance because some groups may not be observed and/or some individuals within groups may not be observed (Graham & Bell, 1989). Our task is to account for this imperfect observation of animals. We chose to address this problem with Bayesian methods both for convenience and to facilitate hierarchical modeling that can account for spatial and temporal variation in abundance (Chelgren, Adams, Bailey, & Bury, 2011; Moore & Barlow, 2011).

Given certain assumptions, we can use distance data to account for imperfect detection of groups (Buckland et al., 2001; Burnham, Anderson, & Laake, 1980). Under distance sampling, we expect a negative relationship between distance from the transect and detection probability of groups. If we specify a detection function, *g*(*d*; θ), we can estimate parameter values, θ, from distance data for *n* groups, $d_{1},d_{2},\ldots ,d_{n}$. Typically, estimating detection parameters relies on the assumption that *g*(*d *=* *0) = 1 (although double‐observer data allow us to relax this assumption, see below).

We developed the model likelihood using a data augmentation approach (Royle, Dorazio, & Link, 2007; see Kéry & Royle, 2016, ch. 8). Under this approach, we augment the observed data set with a large number of unobserved groups that are missing distance and group‐size data. We then estimate a data augmentation parameter, *Ω*, which is the probability that the unobserved groups belong to the sampled population. By fixing the size of the data set, data augmentation simplifies Bayesian analysis of the model by Markov chain Monte Carlo (MCMC), especially when individual or group‐level covariates, such as distance and group size, are used.

The process model in distance sampling rests on the assumption that groups are uniformly distributed in space (or appropriately modeled with covariates). Thus, the process model can be stated by specifying that the distance between groups and the transect is uniformly distributed out to a distance *w* and that membership in the surveyed population of groups in the augmented data set, $z_{i}$, is Bernoulli distributed with probability *Ω*:

*d*_*i*_ ~ uniform (0,*w*)

*z*_*i*_ ~ Bernoulli (*Ω*)

where *z*_*i*_ = 1 if the group belongs to the population and 0 otherwise. If we adopt a half‐normal model for the detection function (uniform, hazard, or other functions are also possible), then the observation model can be specified by stating that the detection of groups is Bernoulli distributed, with probability *p*_*i*_:

$$p_{i}=z_{i}\times e^{-d^{2}/2\sigma^{2}}$$

*y*_*i*_ ~ Bernoulli (*p*_*i*_)

where σ is the shape parameter for the half‐normal distribution and *y*_*i*_ = 1 if the group was detected and 0 otherwise. Under this model, Σ*z*_*i*_ gives the number of groups in the surveyed area.

In a typical application, total abundance would be estimated by multiplying the estimated number of groups by the average observed group size (Buckland et al., 2001; Forsyth & Hickling, 1997). Estimation of both group detection and group size can be refined by including group size as a covariate on detection (Alpizar‐Jara & Pollock, 1996). The process model would then include a distribution for group sizes, with the Poisson distribution being a natural choice (Kéry & Royle, 2016):

*n*_*i*_ ~ Poisson (λ)

where *n*_*i*_ are the observed group sizes and λ is a parameter indicating mean group size. The observation model would then add a group‐size covariate to the shape parameter, so that:

$$\log\left(\sigma_{i}\right)=\beta_{0}+\beta_{1}\log\left(n_{i}\right)\;.$$

If observed group size equals true group size, then Σ*z*_*i*_ *n*_*i*_ is an estimate of total abundance.

We can relax the assumption that detection on the transect is perfect, g(0) = 1, by incorporating double‐observer data into a MRDS model (Borchers et al., 1998; Conn, Laake, & Johnson, 2012; Laake & Borchers, 2004). This extension requires two surveyors to record which groups were observed by each surveyor, generating detection histories for each observed group. In this case, we revise the probability of detection to include the term *p*_*0*_, indicating the probability of detection at *d *=* *0:

$$p_{i}=p_{0}\times z_{i}\times e^{-d^{2}/2\sigma^{2}}.$$

With a single observer, *p*_*0*_ and *Ω* are confounded, but with observations repeated across *i* groups and *j* observers, *y*_*i,j*_, where

*y*_*i,j*_ ~ Bernoulli (*p*_*i*_),

both parameters can be estimated. While double‐observer data allow more flexibility in the group observation model, abundance estimates still assume that group size is accurately recorded.

To account for uncertainty in group size, we integrate an N‐mixture model with the MRDS setup described above. The N‐mixture model assumes each individual within a group is detected independently (conditional on detection of the group), with probability ≤1. This assumption may be violated if surveyors use mental addition, extrapolation, or “guess‐timation” to count large groups. Using the N‐mixture model, group sizes, *n*_*i*_, become latent variables, instead of observed data. The observed counts from each observer, *c*_*i, j*_, are then part of the observation process, with counts the result of a binomial process with order *n*_*i*_ and probability *r*. Furthermore, because counts are only recorded for detected groups, both the counts, and the estimated group sizes, must be ≥ 1. Accordingly, we model both the abundance process and the observation process with zero‐truncated distributions, which we abbreviate ZT. For this model, the totality of the abundance process model includes three distributions:

*d*_*i*_ ~ uniform (0,*w*)

*z*_*i*_ ~ Bernoulli (*Ω*)

*n*_*i*_ ~ ZTPoisson (λ)

where *d*_*i*_ are distance observations, and the data augmentation variable, *z*_*i*_, and group size, *n*_*i*_, are latent variables.

If detection probability of individuals within groups (though not for groups) is constant across distances, the observation process includes two distributions describing the detection of groups and individuals:

$$\log\left(\sigma_{i}\right)=\beta_{0}+\beta_{1}\log\left(n_{i}\right)$$

$$p_{i}=p_{0}\times z_{i}\times e^{-d^{2}/2\sigma^{2}}$$

*y*_*i,j*_ ~ Bernoulli (*p*_*i*_)

*c*_*i,j*_ ~ ZTBinomial (*r,n*_*i*_)

where *y*_*i,j*_ is 1 if observer *j* detects group *i*, and 0 otherwise, and *c*_*i,j*_ gives the observed count if group *i* is detected by observer *j* and is unobserved otherwise. Alternatively, if we expect detection of individuals within a group to decline with distance, we could revise the count model so that:

$$r_{i}=r_{0}\times e^{-d^{2}/2\tau^{2}}$$

*c*_*i,j*_ ~ ZTBinomial (*r*_*i*_ *,n*_*i*_)

where *r*_*0*_ is the probability of detecting an individual on the transect and τ is the shape parameter for the half‐normal detection model. Thus the model may accommodate distance‐based detection in both the observation of the group and of individuals within the group.

To ensure that the above model is identifiable and to compare performance to an MRDS model that assumes group sizes are correctly recorded, we conducted a simulation study. We simulated data under the proposed MRDS‐Nmix model using a variety of parameter settings. In each simulation, there were a total of 200 groups available for detection and survey strip‐width was 100 m. We varied the group size parameter (λ), the effect of distance on detecting a group of size 1 ($\beta_{0}$), the effect of group size on detection ($\beta_{1}$), the effect of distance on detecting individuals (τ), the probability of detecting a group at distance 0 (*p*_*0*_), and the probability of detecting an individual at distance 0 (*r*_*0*_), for a total of 12 simulation scenarios (Table 1). The particular scenarios considered were intended to yield moderate mean detection probabilities (Table 1) because high detection probabilities generate little bias while low detection probabilities require extensive surveys. Furthermore, parameter values were selected so that detection probabilities were high (>0.65) for groups of four animals and individuals within detected groups at 10 m, and low (<0.65) for groups of four animals and individuals within detected groups at 100 m so that the survey strip was not too limited or expansive. Note that our simulations did not include additional unmodeled detection heterogeneity, although this can be an issue in field surveys (Laake et al., 2008). As such, the simulated data exhibit “full independence” (Laake & Borchers, 2004).

**Table 1:** Scenarios used for data simulation and analysis

Scenario	λ	β_*0*_	β_*1*_	τ	*p*_*0*_	*r*_*0*_	$\overset{¯}{p}$	$\overset{¯}{r}$
1	1	ln (65)	0.25	80	1.0	1.0	0.75	0.90
2	1	ln (35)	0.25	40	1.0	1.0	0.48	0.84
3	1	ln (65)	0.25	80	0.8	1.0	0.60	0.90
4	1	ln (45)	0.75	80	0.8	1.0	0.59	0.89
5	4	ln (45)	0.25	80	1.0	1.0	0.69	0.85
6	4	ln (25)	0.25	40	1.0	1.0	0.43	0.76
7	4	ln (60)	0.25	80	0.8	0.9	0.61	0.76
8	4	ln (20)	0.75	80	0.8	0.9	0.62	0.85
9	20	ln (20)	0.35	80	1.0	1.0	0.75	0.84
10	20	ln (13)	0.35	50	1.0	1.0	0.46	0.81
11	20	ln (25)	0.35	80	0.8	0.9	0.60	0.75
12	20	ln (8)	0.75	80	0.8	0.9	0.61	0.75

Parameters include λ: mean size of groups, β_*0*_: the effect of distance on detecting a group of size 1, β_*1*_: the effect of group size on detection, τ: the effect of distance on detecting individuals, *p*_*0*_: the probability of detecting a group at distance 0, and *r*_*0*_: the probability of detecting an individual at distance 0. Resulting mean probability of group detection ($\overset{¯}{p}$) and mean probability of individual detection, given group detection ($\overset{¯}{r}$) are also presented.

For each parameter set, and for each simulated data set, we estimated abundance using two models: one that included an observation model for individual detection and one that did not. We then repeated the simulation and estimation process 200 times for each scenario. For our proposed model and the reduced model, we used the simulations to calculate bias, coverage, and root mean square error for the estimates of total abundance. We simulated data in Program R (v 3.1.1, R Core Team, 2016). We evaluated model likelihoods in JAGS (v 4.2, Plummer, 2003; note that v 4.2 or later is *required* for truncation) using the jagsUI (v 1.3.7, Kellner, 2016) interface in Program R.

## 3 RESULTS

In the 12 scenarios considered, abundance estimates under the MRDS‐Nmix model had lower bias and root mean square error and coverage closer to the nominal rate of 95% than estimates under the standard MRDS model (Table 2). Unsurprisingly, the differences between the models were larger for lower individual detection probabilities. Because individual detection must be high for small groups (due to the zero‐truncation), bias was low for both models for small groups. When group detection probabilities were low, estimates of individual detection, and therefore abundance, were less precise under the MRDS‐Nmix model because there were fewer groups with multiple counts. Overall, parameters were identifiable and bias was reduced by accounting for imperfect detection of individuals.

**Table 2:** Bias, coverage, and root mean square error (RMSE) for total abundance estimates under a mark‐recapture‐distance sampling (MRDS) model and an MRDS‐Nmix model. See Table 1 for description of scenarios

Scenario	MRDS‐Nmix			MRDS model		
	Bias (%)	Coverage (%)	RMSE	Bias (%)	Coverage (%)	RMSE
1	1	86	20	−7	52	27
2	−1	97	30	−14	50	49
3	3	95	19	−5	88	22
4	2	99	23	−8	85	31
5	1	89	48	−13	23	114
6	2	92	78	−21	18	182
7	3	90	69	−19	6	161
8	2	97	78	−20	13	169
9	0	95	220	−15	19	616
10	2	100	315	−19	30	794
11	2	97	259	−23	1	956
12	1	95	267	−24	0	983

## 4 DISCUSSION

Many species of conservation interest reside in groups, including ungulates, cetaceans, galliformes, primates, and others. Surveys for these species often attempt to account for imperfect detection of groups using distance‐sampling or double‐observer methods, but they almost universally assume that detection of individuals within groups is perfect (Conroy et al., 2008; Forsyth & Hickling, 1997; Griffin et al., 2013; Laake et al., 2008). This assumption is primarily due to a lack of suitable models that can account for this second level of imperfect detection, rather than an expectation that it is correct (Conroy et al., 2008; Laake et al., 2008). The available evidence suggests that detection of individuals within known groups is commonly <1. For example, several studies have compared counts of individuals within groups during surveys against more labor‐intensive counts, which are presumed to be more accurate, to estimate detection rates for individuals. In Great Blue Heron (*Ardea herodias*) colonies that averaged 24 nests/colony, aerial surveys detected 72% of nests detected during ground surveys (Dodd & Murphy, 1995). Similarly, a review of aerial photographs of pastured domestic cattle (*Bos taurus*) and horses (*Equus caballus*) in herds of <50 animals detected 83% of the animals detected during ground surveys (Terletzky & Ramsey, 2016). Under more experimental conditions, the proportion of duck decoys counted during aerial surveys ranged from 10% to 80% depending on the habitat type and density of decoys (Smith, Reinecke, Conroy, Brown, & Nassar, 1995). In another experiment, observers detected an average of 71% of White Ibis (*Eudocimus albus*) models in a simulated environment (Frederick, Hylton, Heath, & Ruane, 2003). Furthermore, some studies that relied on the assumption of perfect detection within groups acknowledged some measurement error occurred (Conroy et al., 2008; Walter & Hone, 2003). While detection of individuals may be higher under more favorable conditions, there is clearly a potential for bias in abundance estimates that rely on an assumption of accurate counts within detected groups.

In our simulations, detection of individuals in detected groups depended on the distance to the transect, but averaged between 75% and 90% in the 12 scenarios (Table 1), roughly similar to the examples noted above. Under the MRDS model, which assumed that group sizes were correctly recorded, abundance estimates were 76%–95% of true abundance (Table 2), reflecting the imperfect counts of individuals. Although the MRDS model does not estimate detection of individuals, it does attempt to adjust observed group sizes to account for the effect of group size on detection. Under the assumption of perfect detection of individuals, we expect observed group size to increase with distance, but we can get the opposite trend when distant individuals are harder to detect. As a result, the MRDS model typically underestimated the effect of distance on group detection in our simulations, which might account for the slight amelioration of bias relative to individual detection rates.

The MRDS‐Nmix model achieved the greatest gains in bias, coverage, and root mean square error relative to the MRDS model when the detection of individuals was low (Table 2). The MRDS‐Nmix model also proved more useful at larger group sizes both because there were more individuals with the potential to be missed and because there was a greater probability of observers recording distinct group‐size counts (Table 2). We expect that low group detection rates could hinder MRDS‐Nmix model convergence because fewer groups would have two observations of group size, and replicate counts provide the information necessary to estimate individual‐level detection probability under the N‐mixture model. We also expect that if very large groups are surveyed in real time, the field‐estimates of group size may no longer approximate a binomial process in which individuals are counted independently. If observers use mental addition, extrapolation, or guess‐timation to estimate the size of large groups, group size may be over‐estimated and our proposed estimator may over‐estimate abundance. In this case, surveyors should consider how the survey protocols can be designed to conform to model assumptions.

We note that our unbiased estimates were facilitated using “clean,” simulated data. In practice, field data may violate model assumptions. In particular, group sizes may be over‐dispersed instead of Poisson‐distributed, as assumed by a standard N‐mixture model (Royle, 2004). Previously, various strategies have been developed for N‐mixture modeling of over‐dispersed data, including adopting negative‐binomial, beta‐binomial, or log‐normal distributions (Joseph, Elkin, Martin, & Possingham, 2009; Martin et al., 2011; Royle, 2004). We anticipate that these modeling approaches could also be adapted to the MRDS‐Nmix model to handle over‐dispersed group sizes. We also note that N‐mixture models assume that group‐size counts are independently obtained (Royle, 2004). However, detections obtained under MRDS or double‐observer designs often lack this independence (Barker, 2008). Design approaches for improving independence among observers (e.g., visual and auditory barriers) may improve estimates. The effect of dependence between counts on abundance estimates may be a topic for future research.

We anticipate that the MRDS‐Nmix model could also help surveyors increase sample sizes and reduce costs. Currently, to meet the assumption of perfect detection of individuals, surveys must expend effort to count all individuals in a group. In an aerial survey, for example, this commonly involves circling the group in question until all animals have been counted (Graham & Bell, 1989). This protocol consumes survey time and may also alter the behavior and location of the target group or nearby groups (Forsyth & Hickling, 1997). The MRDS‐Nmix model could allow surveyors to reduce or eliminate circling, which would allow surveyors to complete more transects or reduce costs. Furthermore, surveyors might find it possible to substitute a cheaper survey platform, such as a fixed‐wing aircraft instead of a helicopter. Again, this could save costs or enable additional transects and larger sample sizes.

The MRDS‐Nmix model we introduced requires three types of data for each observed group: a detection history, perpendicular distance to a transect line, and independent counts of group size. An extensive search for field data for use with the proposed model did not uncover a data set for use with this model. We believe that observers typically either record a single consensus group size (Graham & Bell, 1989) or record independent groups sizes and then discard the lower count (Conroy et al., 2008), thereby precluding application of the MRDS‐Nmix model. Although such triple‐sampling data were not readily available, we believe that it is nonetheless practical. Currently, multiple‐observer surveys that record detection histories, group size, and assorted covariates are widely used to monitor ungulates (Griffin et al., 2013), birds (Conroy et al., 2008), marine mammals (Pollock, Marsh, Lawler, & Alldredge, 2006), and other taxa (Fewster & Pople, 2008) via aerial surveys, as well as terrestrial surveys (Kissling & Garton, 2006) and ship‐based surveys (Buckland, Laake, & Borchers, 2010). The primary change required to implement the MRDS‐Nmix model would be to replace a single count of group size with independent counts of group size. We anticipate that this would not be unduly burdensome for most survey teams. Some survey protocols might also need to add distance‐to‐transect data to the survey covariates to implement the full MRDS‐Nmix model. In cases where distance data are judged to be infeasible due to steep terrain or other factors (Conroy et al., 2008; Griffin et al., 2013), it would be relatively simple to reduce the model to a MR‐N‐mixture model by omitting the distance component.

It is well known that double‐observer models are subject to heterogeneity‐induced bias (Barker, 2008). In recent years, authors have combined double‐observer models with other models to try to reduce this bias, creating an MRDS model (Borchers et al., 1998) and a hybrid sightability model (Griffin et al., 2013). The MRDS‐Nmix model (or a reduced MR‐N‐mixture model) is distinct from these previous approaches because it is the first to relax the assumption of perfect detection of individuals. Given that MRDS data are widely collected for grouped animals (Burt et al., 2014), we suggest that the proposed model could be of great practical benefit to science and conservation of wildlife. We hope that the proposed MRDS‐Nmix model will encourage the revision in survey protocols to retain data needed to improve abundance estimates.

## CONFLICT OF INTEREST

None declared.

## DATA ACCESSIBILITY

Computer code for simulating and analyzing data is provided in supporting information.
