# Supplementary material for: Accounting for imperfect detection of groups and individuals when estimating abundance
Source: Ecol Evol. 2017 Aug 8;7(18):7304–10. doi: 10.1002/ece3.3284 (PMC5606903; doi:10.1002/ece3.3284)
Supplement: Supplementary file 5 [file ECE3-7-7304-s005.docx]

Computer code in Supplementary Information will simulate survey data and estimate abundance using two different estimators, MRDS, and MRDS-Nmix. See Clement, Converse and Royle, "Accounting for imperfect detection of groups and individuals when estimating abundance" for details.

NOTE: ****** RUNNING FUNCTION 'mrdsnm.sims' WITH DEFAULT SETTINGS [i.e., mrdsnm.sims()] WILL TAKE APPROXIMATELY ***1 MONTH*** TO COMPLETE ON i5-4210U PROCESSOR WITH 8GB RAM

The code requires Program R, JAGS 4.2 or later, and the jagsUI R package.

Instructions for using code:

1) "four_DO_models.R": save file in an appropriate location.

change the pathname in that file to indicate destination of jags likelihoods

run the code in Program R to write the jags likelihoods

2) save "group_count_data.R" and "MRDSNM sims 170317.R" to an appropriate location

3) change the two 'setwd' commands in “MRDSNM info and examples.R” so that they indicate the location of “group_count_data.R” and the jags likelihoods

4) install R, JAGS, and R package jagsUI, if needed

5) run ‘mrdsnm.sims’ function as described in “MRDSNM info and examples.R”
